# Supplementary figures and images for: Applicability and safety of discontinuous ADVanced Organ Support (ADVOS) in the treatment of patients with acute-on-chronic liver failure (ACLF) outside of intensive care
Source: PLoS One. 2021 Apr 1;16(4):e0249342. doi: 10.1371/journal.pone.0249342 (PMC8016329; doi:10.1371/journal.pone.0249342)

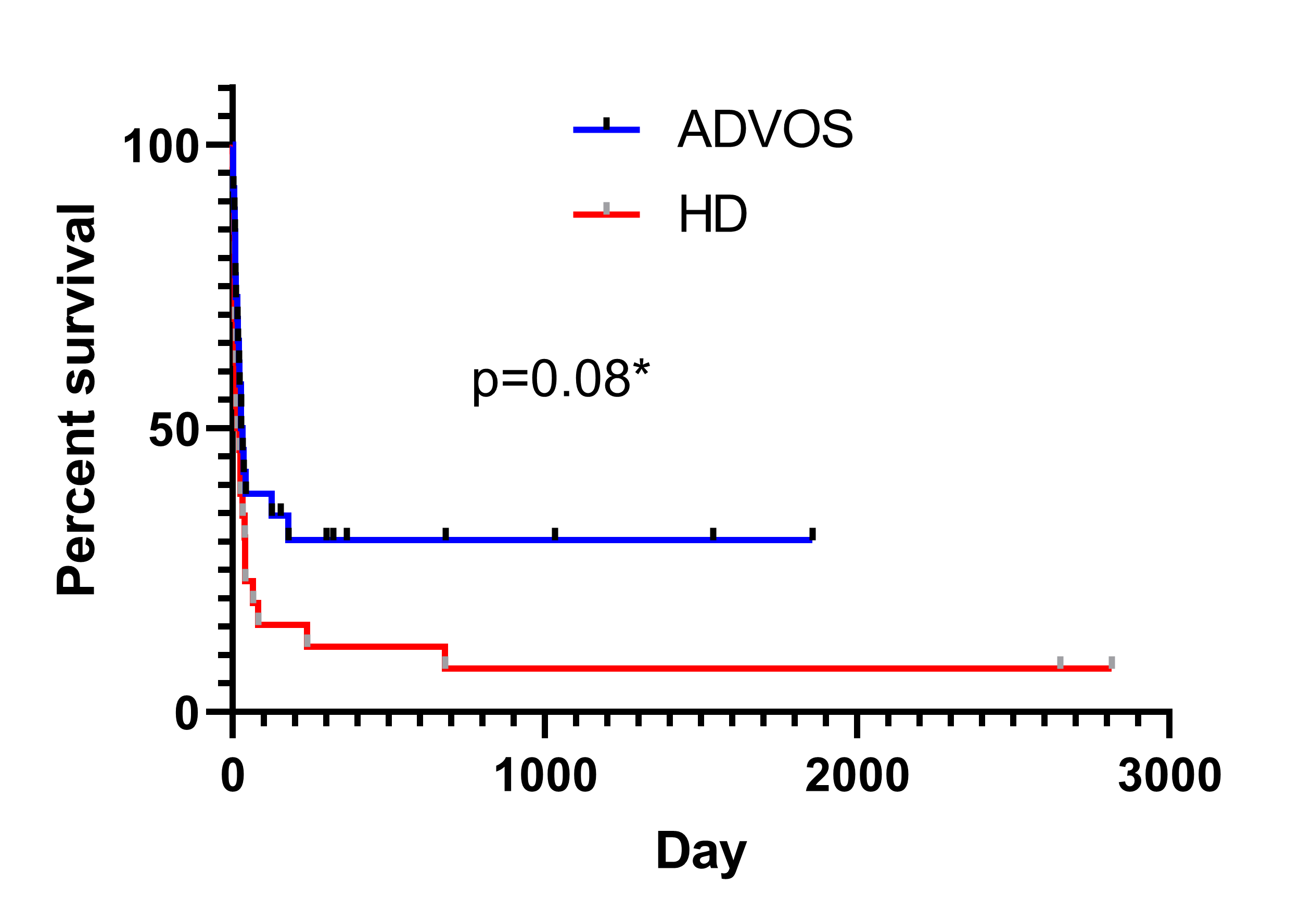

Supplement: S1 Fig — (TIF) [file pone.0249342.s001.tif]
