# Supplementary material for: Applicability and safety of discontinuous ADVanced Organ Support (ADVOS) in the treatment of patients with acute-on-chronic liver failure (ACLF) outside of intensive care
Source: PLoS One. 2021 Apr 1;16(4):e0249342. doi: 10.1371/journal.pone.0249342 (PMC8016329; doi:10.1371/journal.pone.0249342)
Supplement: S1 Table — (DOCX) [file pone.0249342.s002.docx]

**S1 Table. Baseline characteristics of patients of the local ACLF registry, who were used to find appropriate matches for ACLF patients who were treated with ADVOS**

| **Patients, n** | 136 |
| --- | --- |
| Male, n (%) | 89 (65%) |
| Age (years), median (IQR) | 60 (51; 67) |
| Type of liver failure, (%) | ACLF (100) |
|  |  |
| **Etiology of cirrhosis, n (%)** |  |
| Excessive alcohol consumption | 98 (72%) |
| Other or mixed etiology (HBV, NASH, alcohol) | 38 (28%) |
|  |  |
| **Precipitating events as ACLF trigger** |  |
| Infections, n (%) | 37 (28%) |
| Variceal bleeding, n (%) | 38 (28%) |
| Others, n (%) | 61 (44%) |
|  |  |
| **Liver function** |  |
| CLIF-C ACLF Score, median (IQR) | 65 (54.75;73) |
|  |  |
| **Renal failure** |  |
| HRS-AKI, n (%) | 77 (57%) |
| HD treatment days, median (IQR) | 5.5 (2; 9.75) |
