# Supplementary material for: Applicability and safety of discontinuous ADVanced Organ Support (ADVOS) in the treatment of patients with acute-on-chronic liver failure (ACLF) outside of intensive care
Source: PLoS One. 2021 Apr 1;16(4):e0249342. doi: 10.1371/journal.pone.0249342 (PMC8016329; doi:10.1371/journal.pone.0249342)
Supplement: S2 Table — (DOCX) [file pone.0249342.s003.docx]

S2 Table. ADVOS treatment parameters

| UF (ml/h), median (IQR) | 150 (110; 250) |
| --- | --- |
| Blood flow rate (ml/min), median (IQR) | 150 (150; 150) |
| Total UF volume (ml/h) median (IQR) | 1256 (775; 1784) |
