# Supplementary material for: Applicability and safety of discontinuous ADVanced Organ Support (ADVOS) in the treatment of patients with acute-on-chronic liver failure (ACLF) outside of intensive care
Source: PLoS One. 2021 Apr 1;16(4):e0249342. doi: 10.1371/journal.pone.0249342 (PMC8016329; doi:10.1371/journal.pone.0249342)
Supplement: S3 Table — (DOCX) [file pone.0249342.s004.docx]

**S3 Table. Safety parameter before and after the last ADVOS treatment**

| **Safety parameters** | **Prior ADVOS** | **After ADVOS** |
| --- | --- | --- |
| pH, median (IQR) | 7.39 (7.36; 7.42) | 7.41 (7.39; 7.44) |
| Bicarbonate (mmol/l), median (IQR) | 22 (20.45; 23.55) | 25 (22.15; 25.25) |
| Base Excess (mmol/l), median (IQR) | -2.3 (-4.6; -0.75) | -0.45 (-2.1; 1.3) |
| Potassium (mmol/l), median (IQR) | 3.7 (3.4; 4.2) | 3.9 (3.7; 4.2) |
| Sodium (mmol/l), median (IQR) | 138.3 (133; 139) | 135 (133; 139) |
| Arterial pressure, median (IQR) | 69 (63; 81) | 69 (64; 74) |
| Thrombocytes (/nl), median (IQR) | 83 (60; 132) | 85 (51; 91) |
| Prothrombin time-INR, median (IQR) | 2 (1.7; 2.4) | 1.9 (1.6; 2.2) |
