# Supplementary material for: Applicability and safety of discontinuous ADVanced Organ Support (ADVOS) in the treatment of patients with acute-on-chronic liver failure (ACLF) outside of intensive care
Source: PLoS One. 2021 Apr 1;16(4):e0249342. doi: 10.1371/journal.pone.0249342 (PMC8016329; doi:10.1371/journal.pone.0249342)
Supplement: S4 Table — (DOCX) [file pone.0249342.s005.docx]

**S4 Table. Concentration dependent elimination of bilirubin after cumulative 16 h of ADVOS in discontinuous treatment.**

| **Bilirubin before ADVOS (mg/dl)** | **Elimination of toxins after 16 h*** | **p-value^ǂ^** |
| --- | --- | --- |
| Bilirubin ≤ 20 mg/dl, median (IQR) | -2.32 (-9.6; -2.3) | 0.08 |
| Bilirubin ≤ 30 mg/dl, median (IQR) | -9.1 (-8.1; 16.7) |  |
| Bilirubin ≤ 50 mg/dl, median (IQR) | -21.6 (-16.5; 18.3) |  |

*two treatment cycles on consecutive days, while one cycle endured 8 h; ^ǂ^Calculated one-way anova analysis
