# Supplementary material for: Applicability and safety of discontinuous ADVanced Organ Support (ADVOS) in the treatment of patients with acute-on-chronic liver failure (ACLF) outside of intensive care
Source: PLoS One. 2021 Apr 1;16(4):e0249342. doi: 10.1371/journal.pone.0249342 (PMC8016329; doi:10.1371/journal.pone.0249342)
Supplement: Attachment — Submitted filename: Response to Reviewers-2_.docx [file pone.0249342.s006.docx]

**Point-by-point reply**

The revised manuscript has been modified according to the comments and all changes in the former version of the manuscript are highlighted in yellow (Revised Manuscript with Track Changes). To us, the comments and consecutive changes significantly improved the quality of the manuscript and after careful revision we hope that our manuscript can further be considered for publication in PLOS ONE.

**Point-by-point reply to all comments raised by the reviewer**

**Reviewer 1:**

Authors concluded discontinuous ADVOS treatment outperformed hemodialysis in reducing protein-bound metabolites. The data of the article is attractive. However, authors need to be more careful not to mislead. Revisions required are as follows:

Major comments:

1.	There is no significant difference between 28-days survival in ADVOS treated cohort and HD treated cohort. Avoid misleading expressions and keep the content that it was possible to indicate non-inferiority.

*We fully agree and want to avoid overinterpretation of the data. Therefore, we toned the language in our abstract and discussion section down (page 4, 11 and 14).*

2.	Concerning 44% (IQR 30; 85), is 28-days ‘survival’ correct or 28-days ‘mortality’ correct? The description in the text and the description in the table do not match.?

*We have changed 28-days survival to the more appropriate term 28-days mortality in the text and the description of the table.*

3.	It would be better if there is a comparison between predicted 28-days survival in the ADVOS treated cohort and HD treated cohort.

*We agree and performed additional analysis. These results can be found in table 4 point 7. There was* no difference between ADVOS and HD treated patients, suggesting appropriate matching.

Minor comments:

1.	In Abstract, in line 19, the correct one is ‘‘stronger’’, not ‘‘stringer’’.

*Thanks for your thoroughness. We corrected this accordingly.*

2.	In Results, in line 2, the correct one is ‘‘65.3%’’, not ‘‘53.5%’’.

*We corrected this mistake accordingly.*

*3.*	Concerning main etiology of cirrhosis in three patients, is ‘a mixed etiology (HBV, alcohol or NASH)’ correct or ‘viral hepatitis (HBV, HCV)’ correct? The description in the text and the description in the table do not match.

*This was a mistake. We corrected this accordingly.*

*4.*	In Results, in line 8, what is ‘hydropy’?

*We have changed hydropy to fluid overload as the appropriate term.*

5.	In Results, in line 12, and In Table 2 and In Supplementary table 3, the correct one is ‘‘prothrombin time-INR’’, not ‘‘INR’’.

*We corrected this to the correct term.*

**Reviewer 2:**

Major comments:

1.	The authors described in the summary that ‘The 28-days survival rate in the ADVOS group was 56% (14/26) exceeding the expected survival time according to 12% (predicted median 28-days survival was 44%, IQR 30; 85)’ and in the conclusion section ‘we found a trend that ADVOS carries a survival advantage in patients with ACLF. These sentences seem to be sophistical argument. Predicted 28-days survival was calculated in each patient by the CLIF-C ACLF calculator. It should not be described that the 28-days survival rate in the ADVOS group exceeded the expected value by 12%.

The effect of ADVOS on the 28-days survival of patients with ACLF should be assessed statistically according to the result of case control study: 56% (14 of 25) in the ADVOS group vs 40% (10 of 25) in the HD group. Log-rank test is also able to be used for survival analysis.

*We agree and we aimed not to overinterpret our data. We have changed “28-days survival rate in the ADVOS group exceeded the expected value by 12% “ to “The 28-days mortality in the ADVOS group was 56% (14/26) and was not inferior to predicted survival (predicted median 28-days mortality was 44%, IQR 30; 59).” in the abstract (page 4). Additional amendments were made in the results section: “In addition, predicted survival by the CLIF-C ACLF score was 44% (IQR 30; 59, table 2) in the ADVOS cohort, suggesting that ADVOS does not negatively impact survival.” (page 11). And in the conclusion section “we found that ADVOS was not inferior in 28-days morality compared to regular hemodialysis in patients with ACLF, future randomized trials are needed to investigate its prognostic effect compared to regular HD.” (page 14).*

*Following the suggesting of reviewer 2, we have performed a short-term (28-days mortality) (data added to table 4) and long-term survival analysis (supplementary figure 1) between ADVOS vs. HD treated patients, using the appropriate statistical test (T-test or Log-rank test, respectively). Here, we found no significant (p=0.08) difference between the groups. We thank the reviewer for the advice.*

2.	In Table 2, the data of ACLF grade should be shown in numbers of patients belonging to each grade: grade 1, 2, 3, instead of median and IQR.

*We have added the missing data in table 2.*

3.	Urine volume could be assessed before and after treatment with ADVOS and with HD.

*All of the included patients were diagnosed with HRS-AKI and anuria/oliguria as stated in table 1. We have added this information in table 1. Unfortunately, data on urine volume after treatment with ADVOS or HD are not available and we are unable to provide this information.*

4.	The title should be corrected partially: patients with acute liver failure (ACLF)⇒patients with acute-on-chronic liver failure (ACLF).

*Thank you very much for your thoroughness. We corrected this mistake accordingly.*

5.	The authors should ensure that tables are the best format to present their data.

*We have optimized the presentation of the tables for the needed format and hope that the data is well presented. Further, we have added an additional figure in the supplementary section also to ensure a well-balanced presentation of our data between graphs and tables.*

6.	The description of matching criteria 1 in Table 4 is insufficient. How many patients were male?

*We have added this information in table 4.*

Comments from the Editorial Office:

"Supported by internal research funds of the University Medical Centre Mainz"

We note that you have provided funding information that is not currently declared in your Funding Statement. However, funding information should not appear in the Acknowledgments section or other areas of your manuscript. We will only publish funding information present in the Funding Statement section of the online submission form. Please remove any funding-related text from the manuscript and let us know how you would like to update your Funding Statement. Currently, your Funding Statement reads as follows:

"The author(s) received no specific funding for this work."

*We deleted any funding-related text from the manuscript.*
