# Supplementary material for: Applicability and safety of discontinuous ADVanced Organ Support (ADVOS) in the treatment of patients with acute-on-chronic liver failure (ACLF) outside of intensive care
Source: PLoS One. 2021 Apr 1;16(4):e0249342. doi: 10.1371/journal.pone.0249342 (PMC8016329; doi:10.1371/journal.pone.0249342)
Supplement: Attachment — Submitted filename: Response to Reviewers V3.docx [file pone.0249342.s007.docx]

| To the Editors  PLOS ONE | **Prof. Dr. med. Julia Weinmann-Menke, MD**  Director of Nephrology  First medical department  Bldg 605  Langenbeckstrasse 1  55131 Mainz    Phone: +49 (0) 6131 171  Fax: +49 (0) 6131 17477282  E-Mail: julia.weinmann-menke@unimedizin-mainz.de  http://www.unimedizin-mainz.de/1-med |
| --- | --- |
|  | Mainz, 9. März 2021 |

**PONE-D-21-02242 entitled "Applicability and safety of discontinuous ADVanced Organ Support (ADVOS) in the treatment of patients with acute-on-chronic liver failure (ACLF) outside of intensive care"**

Dear Dr. Kanda,

We would like to thank you for returning the reviewer comments and providing us with the opportunity to resubmit after minor revisions. The revised manuscript contains all changes highlighted and a point-to-point response is included below. We hope that you can now accept the manuscript for publication in PLOS ONE.

Sincerely yours,

Julia Weinmann-Menke, MD

**Point-by-point reply**

The revised manuscript has been modified according to the comment and all change in the former version of the manuscript are highlighted in yellow (Revised Manuscript with Track Changes).

**Point-by-point reply to all comments raised by the reviewer**

**Reviewer 2:**

Leonard Kaps et al. revised their manuscript carefully and adequately.

They disclosed that only one patient in the ADVOS cohort received liver transplantation and created supplementary figure1.

Minor comments:

They should describe whether a liver-transplanted patient was censored at the time of transplantation in the long-term survival analysis or not (Log-rank test, supplementary figure 1, p=0.08).

1.	We apologize that this detail escaped our attention and agree that it should be noted whether the transplanted was censored or not in the analysis. We treated the transplanted patient as a complete case (death) and, thus, he was not censored in the analysis. Accordingly, we added the following passage in the methods section “The one patient was transplanted due to terminal hepatic failure and was not censored in the analysis, being treated as a complete case”. Further, we mentioned in the description of supplementary figure 1 “the one patient who received liver transplantation was not censored in the analysis”.
